# Maxillary Anterior Root Position/Angulation and Alveolar Bone Thickness in the Saudi Population: Implications for Implant Therapy

**DOI:** 10.1155/ijod/4469010

**Published:** 2025-02-20

**Authors:** Wesam Fathi, Kadi Alkheraije

**Affiliations:** ^1^Department of Periodontology and Implant Dentistry, College of Dentistry, Qassim University, Buraydah, Qassim, Saudi Arabia; ^2^College of Dentistry, Qassim University, Buraydah, Qassim, Saudi Arabia

## Abstract

**Background:** Immediate implant placement has been considered a rapid and relatively efficient oral rehabilitation method that restores functional and esthetic demands. Understanding the anatomical tooth position and the natural dimensions of the alveolar ridge would facilitate proper treatment planning for immediate implants particularly in the esthetic zone. Therefore, the present study aims to use cone-beam computed tomography (CBCT) images to evaluate sagittal root position (SRP), tooth angulation within the alveolar ridge, and measuring labial and palatal alveolar bone thickness at maxillary anterior teeth.

**Materials and Methods:** CBCT images of 102 Saudi adult subjects were used to evaluate the maxillary anterior teeth for three main parameters: SRP classification, root angulation in the alveolar bone, and labial and palatal bone thickness.

**Results:** A total of 612 teeth were evaluated. Three hundred eighty-three teeth were classified as SRP Class I; majority of canines (75%), 46.08% of lateral incisors and 66.67% of central incisors. For SRP Class I, 46.5% of the teeth belong to females and 53.5% to males. In SRP Class II, 71.1% are female and 28.9% are male. SRP Class III has 33.3% females and 66.7% males. For SRP Class IV, 57.3% are female and 42.7% are male. Males have statistically significant thicker labial bone at canines and lateral incisors, at 2, 4, and 6 mm. However, males have statistically significant thicker bone at central incisors in palatal measurements and at the apex.

**Conclusion:** There are significant differences in how canines, lateral, and central incisors are distributed across SRP classifications (*p*  < 0.001). Canines show high frequency in Class I, but low in Class II. Lateral incisors have more even distribution between Class I and II. Central incisors follow similar pattern to canines with high Class I. The variations in SRP class and the gender differences in bone thickness identified in the current study confirm the necessity of personalized treatment plans to enhance immediate or even delayed implant placement success rates.

## 1. Introduction

In oral rehabilitation, immediate implant placement has been described as a successful functional and esthetic treatment. Achieving a favorable result requires the creation of a healthy and natural-appearing supra-implant gingival morphology with the proper volume, color, and shape [1]. However, empirical research has demonstrated that the resorption of buccal bone and subsequent dimensional changes in the alveolar ridge that occur after tooth extraction are not halted by immediate implant placement. Research has indicated that remodeling has a greater impact on the facial (labial/buccal) bony plates compared to the lingual (palatal) plates. Furthermore, resorption is more pronounced on the horizontal aspect of the bony plates than on their vertical counterparts. Due to the compromise of the facial bony plates caused by the remodeling process, immediate implant placement is complicated [2]. The evaluation of sagittal root position (SRP) in relation to the anterior maxillary osseous housing for immediate implant placement is a crucial aspect of treatment planning. Kan et al. [3] and Kong, Kim, and Lee [4] both provide classification systems for this evaluation, with Kan et al. [3] emphasizing the clinical relevance and Kong highlighting the need for cone-beam computed tomography (CBCT) analysis. Moreover, Moghaddas and Behravan[5] depending on CBCT images had introduced a new classification of sagittal root positioning of root apex for mandibular teeth. Tao et al.[6], Medikeri et al. [7], and Soumya et al. [8] further contribute to this area by investigating the anatomical aspects of the maxillary anterior teeth and the prevalence of labial bone perforations, respectively. However, there is a knowledge gap in the literature regarding the specific challenges and considerations in evaluating SRP for immediate implant placement, particularly in different patient populations. Further research is needed to address this gap and enhance the understanding of this important aspect of treatment planning. Therefore, the aim of the study is to evaluate SRP, angulation between the tooth axis and alveolar bone axis within the anterior alveolar arch, width of alveolar bone labially, and palatally in the anterior esthetic region using CBCT images.

### 1.1. Clinical Relevance

The current study offers a better understanding of the normal angulation of maxillary anterior teeth and the natural dimensions of the alveolar ridge around them. As patients would prefer immediate replacement of a hopeless tooth, clinicians should be familiar with the approximate alveolar ridge thickness and its correlation with the natural tooth. Accordingly, an implantologist would precisely plan an immediate implant placement within the extraction socket with the highest possible success rates.

## 2. Material and Methods

This retrospective cross-sectional study was approved by the Committee of Research Ethics, Deanship of Scientific Research, Qassim University, Saudi Arabia (No. 24-90-02). CBCT images of the anterior maxillary region were analyzed for the study. It included 102 Saudi patients who met the following inclusion criteria: Saudi nationality, at least 18 years old at the time of the CBCT scan, fully developed and intact maxillary incisors and canines, no evidence of periapical pathology, and no history of surgical treatment (guided bone/tissue regeneration) or orthodontic treatment in the anterior maxillary dentition.

### 2.1. SRP Classification

**Figure 1:**
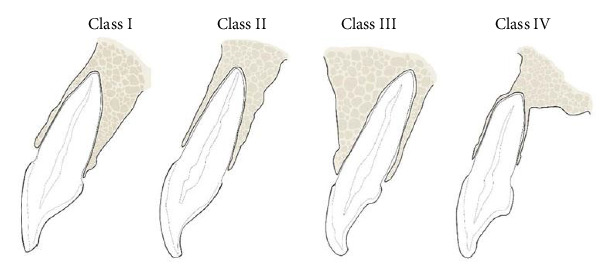
Sagittal root position (SRP) classification by Kan et al. [3 ].

The CBCT images have undergone evaluation to determine the correlation between the SRP of the maxillary anterior teeth and their corresponding osseous housing. This correlation was documented and classified as Class I, II, III, or IV. The classification used was SRP classification by Kan et al. [3]. Each SRP in relationship to its osseous housing was classified as follows (Figure 1):

Class I: The root is positioned against the labial cortical plate.

Class II: The root is centered in the middle of the alveolar housing without engaging either the labial or the palatal cortical plates at the apical third of the root.

Class III: The root is positioned against the palatal cortical plate.

Class IV: At least two thirds of the root is engaging both the labial and palatal cortical plates.

**Figure 2:**
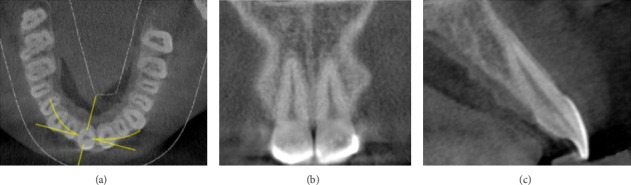
Three-dimensional guide for analysis. (A) Axial view. (B) Coronal view. (C) Sagittal view.

CBCT image for each tooth can be viewed through axial, coronal, and sagittal views (Figure 2). The sagittal view is the one used for recording: SRP classification, root angulation within the alveolar bone, and labial and palatal bone thickness.

### 2.2. Angle of Roots in the Alveolar Bone

**Figure 3:**
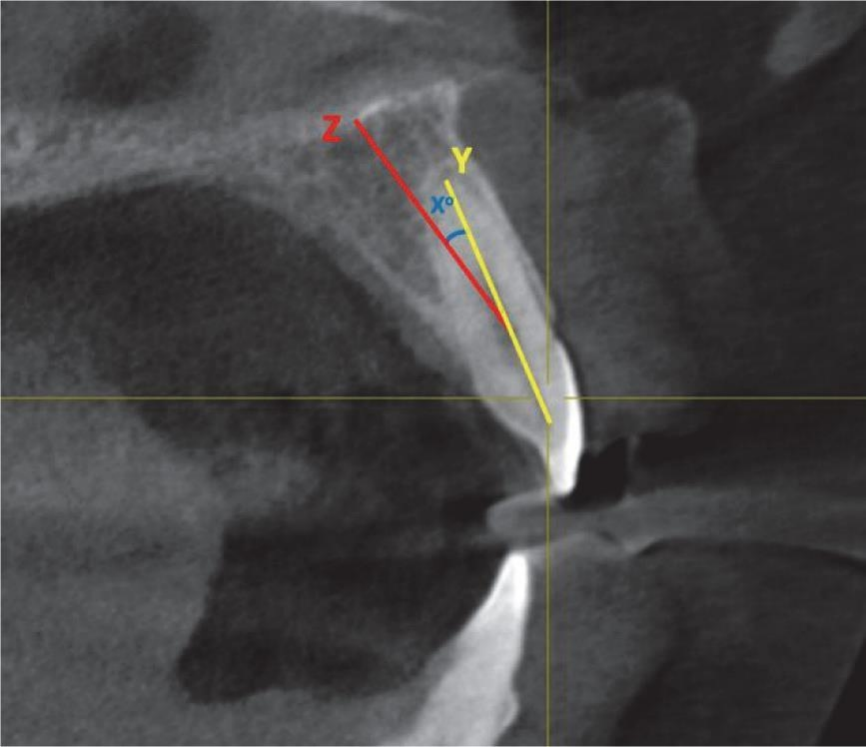
*X* ^o^ is the tooth angulation within the alveolar ridge. *X* is the angle between *Y*-line (tooth long axis) and *Z*-line (alveolar ridge axis).

The angulation of the roots within the alveolar bone was assessed by measuring the angle between the longitudinal axes of the anterior teeth and the alveolar bone. To do this, the midpoints of the buccolingual alveolar bone were identified in the crestal region, then connected to a mid-point at the base of alveolar bone; alveolar bone axis. A line was drawn from the root apex to the pulp horn to represent the longitudinal axis of the anterior tooth; tooth longitudinal axis. The angle between this line and the alveolar bone axis was then measured. This analysis was performed on the right and left maxillary central incisors, maxillary lateral incisors, and maxillary canines (Figure 3).

### 2.3. Labial and Palatal Bone Thickness

**Figure 4:**
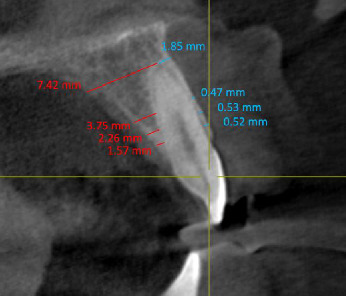
Measures of labial and palatal bone thickness at 2, 4, and 6 mm from crest and at the apex labially and palatally.

The thickness of the labial and palatal bones was measured at four points perpendicular to the longitudinal axis of the maxillary incisors and canines, at distances of 2, 4, and 6 mm from the alveolar crest and at root apex (Figure 4).

All measurements were performed by the same examiner. To ensure reliability, intraexaminer calibration was conducted by measuring 5 records (SRP, root angulation, and labial bone thickness at 2, 4 , and 6 mm from the alveolar crest) from randomly selected images of 30 teeth on two separate days, yielding an intraclass correlation coefficient (ICC) of 0.7.

The data were analyzed using SPSS, with a significance level set at *p*  < 0.05. The *χ*^2^ test was used to check the association between categorical variables, such as gender (independent variable) and SRP classifications or other classifications by tooth type (dependent variables). For continuous variables like thickness measurements, *t*-tests were used to compare the means between groups. When any cell in the *χ*^2^ test had an expected count of less than 5, Fisher's exact test was used instead.

## 3. Results

In this study, a total of 612 teeth were evaluated to determine various parameters related to SRP classifications, teeth angulations, and bone thickness measurements.

Table 1 shows the demographic distribution of the study participants in the study. The mean age of the participants is 35.49 years, indicating that, on average, the sample is comprised of middle-aged adults. The standard deviation (SD) is 11.81, reflecting the variability in the ages of the participants and suggesting a diverse age range within the sample. The minimum age recorded is 19 years, while the maximum age is 68 years.

### 3.1. SRP Classifications

Table 2 shows the frequency distribution of SRP classification for various tooth types shows distinct patterns.

**Figure 5:**
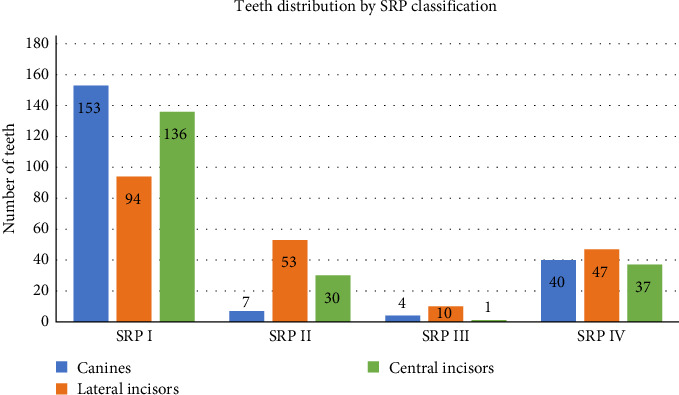
Distribution of sagittal root position (SRP) classification for canines, lateral incisors, and central incisor.

Table 3 and Figure 5 show the distribution of SRP classification for canines, lateral incisors, central incisors, and the overall distribution reveals distinct patterns and trends across the different tooth types.

For canines, the majority of cases are classified as SRP I. This is followed by SRP IV, SRP II, and SRP III, respectively. The high prevalence of SRP I classification suggests that the canines are most frequently positioned in the ideal alignment, with fewer cases showing deviations as indicated by the lower numbers in SRP III, II, and IV classifications.

The lateral incisors display a different pattern, with the highest frequency in SRP I. This is followed by SRP II, SRP IV, and SRP III. The distribution shows a more balanced spread among the classifications, with a significant proportion of lateral incisors falling into SRP II and SRP IV categories, indicating more variability in the root positions compared to canines.

Central incisors predominantly fall into the SRP I category, followed by SRP IV, SRP II, and SRP III. This distribution is similar to that of canines, with a strong preference for the SRP I classification, but it also shows a notable presence in SRP IV and II, suggesting some degree of positional variability.

Based on the *χ*^2^ analysis, there are significant differences in how the three types of teeth are distributed across SRP classifications (*p*  < 0.001). Canines show high frequency in Class I (153), but low in Class II (7). Lateral incisors have more even distribution between Class I (94) and II (53). Central incisors follow similar pattern to canines with high Class I (136).

### 3.2. Tooth Angulations

**Figure 6:**
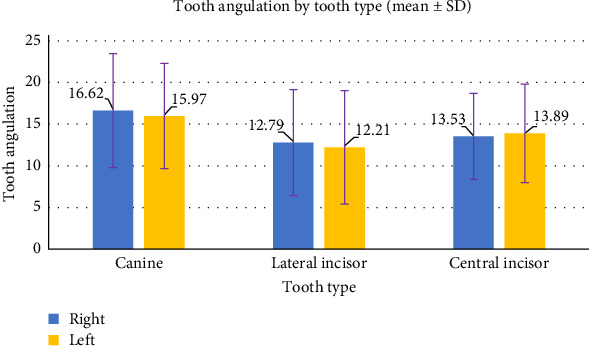
Descriptive statistics of tooth angulation by tooth type.

Table 4 presents the descriptive statistics of the angle by tooth type for both the right and left sides. For the right side, the canine teeth show the highest mean angle of 16.62°. The lateral incisors have the least angle of 12.79°. The central incisors on the right side have a mean angle of 13.53°. On the left side, the central incisors have a mean angle of 13.89°. The lateral incisors exhibit the least mean angle of 12.21°. The canine teeth on the left side have the highest mean angle of 15.97° (Figure 6).

### 3.3. Alveolar Bone Thickness

Tables 5 and 6 present descriptive statistics for labial and palatal bone thickness, respectively.

### 3.4. Relation Between Different Variables

The association between gender and SRP classification with each tooth type is analyzed in Table 7. Meanwhile, Table 8 shows the distribution of SRP classification of teeth according to gender.

Table 9 compares the thickness means of canines by gender at various distances from the crest and at the apex, for both labial and palatal measurements. For labial thickness, males exhibit significantly higher mean values than females at 2 mm from the crest, 4 mm from the crest, and 6 mm from the crest. However, at the apex, the difference is not statistically significant. For palatal thickness, males also have significantly higher mean values at 2 mm from the crest, 4 mm from the crest, 6 mm from the crest, and at the apex.

Table 10 presents the comparison of lateral incisors thickness means by gender at various distances from the crest and at the apex for both labial and palatal measurements. For labial thickness, males have significantly higher mean values than females at 2 mm from the crest, 4 mm from the crest, and 6 mm from the crest. However, at the apex, the difference is not statistically significant. For palatal thickness, males also show significantly higher mean values than females at 2 mm from the crest, 4 mm from the crest, 6 mm from the crest, and at the apex. These results indicate that males generally have thicker bone at lateral incisors compared to females at all measured distances, with statistically significant differences observed at most measurement points except for the labial measurement at the apex.

Table 11 compares the thickness means of central incisors by gender at various distances from the crest and at the apex, for both labial and palatal measurements. For labial thickness, the differences between males and females are not statistically significant at 2 mm from the crest, 4 mm from the crest, and 6 mm from the crest. However, at the apex, males have a significantly higher mean thickness than females.

For palatal thickness, males consistently show significantly higher mean values compared to females at all measured distances. The measures indicate that, while there are no significant gender differences in labial thickness at most distances, males generally have thicker bone at central incisors in palatal measurements and at the apex, with statistically significant differences.

## 4. Discussion

The current study aimed to evaluate the thickness of the buccal and palatal alveolar bone and the angle of the roots in the anterior esthetic region using CBCT reports. Several studies had used CBCT to evaluate bone thickness at sites of future dental implants, especially in the esthetic zone. While Nowzari et al. [9] evaluated facial alveolar bone thickness at maxillary central incisors, the present study investigated the whole anterior teeth for a more comprehensive evaluation. Moreover, the present study evaluated not only the horizontal dimension, labially and palatally, of alveolar bone housing of a future implant, but also sagittal tooth position within the alveolar ridge.

The distribution of SRP classifications in the current study revealed that the majority of roots were positioned against the labial cortical plate (SRP I: 62.6%). This finding aligns with Tao et al. [6], who also reported a predominance of SRP I in their sample. Kan et al. [3] similarly found a significant number of SRP I classifications, followed by SRP IV classifications, which is consistent to the current study. The measurements of labial and palatal bone thickness in the current study were consistent with those reported by Kong, Kim, and Lee [4], Soumya et al. [8], and Alqhtani et al. [10], who emphasized the critical role of CBCT in accurately assessing bone dimensions. For instance, findings that labial bone thickness decreases significantly as one moves apically are in line with their observations. This consistency across studies underscores the reliability of CBCT as a tool for evaluating bone thickness, crucial for planning successful implant placements.

The mean angles of different tooth types measured in the current study showed strong correlations with those reported by Medikeri et al. [7] and Tao et al. [6]. For example, the mean angle for the right canine in the current study was 16.62°, which is comparable to the findings of Medikeri et al. [7]. The minor variations in angles could be attributed to differences in measurement techniques or the anatomical characteristics of the sample populations. Significant differences in bone thickness between genders were observed in the current study, with males generally exhibiting thicker bone measurements. This finding is consistent with the results of Kong, Kim, and Lee [4] and Medikeri et al. [7], who also reported gender-based variations in bone thickness. These differences highlight the importance of considering gender when planning implant placements, as it may influence the stability and success of the implants.

Rodrigues et al. [1] and Naiem, Shujaat, and Shah [2] provided insights into the relationship between tomographic SRP and bone housing, findings that were corroborated by the current study. Their research, which highlighted the importance of CBCT in visualizing root positioning and bone thickness, supports the conclusions regarding the efficacy of CBCT in implant planning. Similarly, the anatomical aspects investigated by Tao et al. [6] and the prevalence of labial bone perforations reported by Medikeri et al. [7] were consistent with the current observations. Moghaddas and Behravan [5] introduced a new classification of sagittal root positioning, finding higher rates of SRP III in their sample. This contrasts with the current findings, where SRP III was the least common classification. Such discrepancies emphasize the need for further research to explore these variations across different populations and clinical scenarios. Additionally, the studies by Al-Nawas, Schiegnitz, and Kammerer [11] and Tawfik, El-Beialy, and Ali [12] on immediate implant placement and bone remodeling align with the observations of buccal bone resorption and its impact on implant success.

Using CBCT for measuring the inclination of healthy maxillary anterior teeth, Han et al. [13] had found that the long axis of anterior teeth is more inclined than the alveolar process. Further comparisons can be drawn with studies by Ferreira et al.[14] and Petersen et al. [15]. Ferreira et al. [14] explored the relationship between root angulation and implant success, finding that proper angulation significantly affects implant stability. This supports the current study's emphasis on the importance of accurately measuring root angles for successful implant planning. Petersen et al. [15] examined the impact of alveolar bone thickness on the longevity of dental implants. Their findings that thicker alveolar bone correlates with longer implant survival are consistent with the current study's observations of the importance of bone thickness in implant success. Barboza, Monteiro, and Cavalcanti [16] studied the effects of gender differences on dental implant outcomes and found significant variations similar to those in the current study. Their research indicated that males generally have thicker bone, which might contribute to higher implant success rates. This aligns with the current findings and underscores the need for gender-specific treatment plans. The study by Naiem, Shujaat, and Shah [2] on the variability of SRP classifications in different populations further supports the current study's observations. They found that anatomical variations significantly impact SRP classifications, which aligns with the current study's finding of a high prevalence of SRP I.

Comparing the current study's findings with Alqerban et al. [17] and Zekry et al. [18] reveals further consistencies and variations. Alqerban et al. [17] assessed alveolar bone thickness in relation to age and gender in a Syrian population and found that males had significantly thicker bone measurements compared to females, mirroring the current study's findings. Additionally, their study noted variations in bone thickness across different age groups, which the current study did not extensively explore but suggests could be an important area for future research. Zekry et al. [18] investigated labial bone thickness in the anterior maxilla of an Egyptian population using CBCT. They reported that the labial bone thickness decreases apically, consistent with the current study's observations. However, Zekry et al. [18] also found regional differences in bone thickness, suggesting that geographical and ethnic factors may play a significant role in bone morphology.

The consistent trends across studies reinforce the importance of CBCT in implant planning, particularly in assessing bone thickness and root positioning. The current study emphasizes the importance of accurate CBCT interpretation prior to immediate implant placement, especially, in the esthetic zone. Clinicians must be aware of different tooth angulations and limited alveolar ridge dimensions around maxillary anterior teeth when planning to offer their patients immediate implants.

The gender differences identified in the current study underscore the need for personalized treatment plans to enhance implant success rates. Furthermore, the variations in SRP classifications and bone thickness measurements highlight the importance of considering anatomical differences when planning implants.

Despite the valuable insights gained, the current study has several limitations that must be acknowledged. The study did not account for other factors that could influence bone thickness and root positioning, such as the presence of periodontal disease or prior dental treatments. While gender differences were explored, other demographic variables such as lifestyle factors and genetic predispositions were not thoroughly examined, which could provide a more comprehensive understanding of the influences on alveolar bone and root angulation. Addressing these limitations in future research could enhance the applicability of the findings.

## 5. Conclusion

Within the limitations of this study, significant differences were observed in the prevalence of SRP classes among maxillary anterior teeth. These variations in SRP class frequencies highlight the necessity of evaluating each maxillary anterior tooth individually during CBCT exams as part of the decision-making process for immediate or even delayed implant placement.

## Figures and Tables

**Table 1:** Frequency distribution of the demographic variables.

Demographic variable	*n*	%
Gender		
Female	53	52
Male	49	48
Age in years		
18–29	37	36.3
30–44	47	46.1
45 +	18	17.6
Total	102	100

**Table 2:** Frequency distribution of sagittal root position (SRP) classification by tooth type.

Side	Tooth type	SRP classification			
		I	II	III	IV
Right	Canine	78 (76.5)	4 (3.9)	2 (2)	18 (17.7)
	Lateral incisor	49 (48)	29 (28.4)	6 (5.9)	18 (17.7)
	Central incisor	70 (68.6)	14 (13.7)	0 (0)	18 (17.7)

Left	Central incisor	66 (64.7)	16 (15.7)	1 (1)	19 (18.6)
	Lateral incisor	45 (44.1)	24 (23.5)	4 (3.9)	29 (28.4)
	Canine	75 (73.5)	3 (2.9)	2 (2)	22 (21.6)

**Table 3:** Canines, lateral incisors, central incisors, and overall distribution by sagittal root position (SRP) classification.

Tooth type	SRP classification				Total
	I	II	III	IV	
Canines	153 (75)	7 (3.43)	4 (1.96)	40 (19.61)	204
Lateral incisors	94 (46.08)	53 (25.98)	10 (4.9)	47 (23.04)	204
Central incisors	136 (66.67)	30 (14.70)	1 (0.49)	37 (18.14)	204
Overall SRP	383 (62.58)	90 (14.71)	15 (2.45)	124 (20.26)	612

**Table 4:** Descriptive statistics of angle by tooth type.

Side	Tooth type	Mean	SD	Minimum	Maximum
Right	Canine	16.62	6.84	3.8	33.6
	Lateral incisor	12.79	6.36	1.8	35.5
	Central incisor	13.53	5.16	2.8	26.6

Left	Central incisor	13.89	5.91	3	31
	Lateral incisor	12.21	6.82	2.3	40
	Canine	15.97	6.32	4.7	36.8

Abbreviation: SD, standard deviation.

**Table 5:** Descriptive statistics of labial bone thickness by tooth type

Toothtype	Distance	Mean	SD	Minimum	Maximum
Right canine	2 mm below crest	1.1	0.56	0.12	3.45
	4 mm below crest	1.12	0.56	0.34	3.26
	6 mm below crest	0.99	0.5	0.08	3.4
	At apex	2.15	1.1	0.1	4.56

Right lateral incisor	2 mm below crest	1.1	0.47	0.3	2.51
	4 mm below crest	1.19	0.64	0.16	3.07
	6 mm below crest	1.05	0.58	0.09	2.8
	At apex	2.38	1.16	0.09	6.45

Right central incisor	2 mm below crest	1.05	0.32	0.38	2.41
	4 mm below crest	1.06	0.37	0.34	2.19
	6 mm below crest	0.94	0.37	0.19	2.34
	At apex	1.9	0.85	0.09	4.1

Left central incisor	2 mm below crest	1.06	0.32	0.09	1.9
	4 mm below crest	1.09	0.37	0.16	2.16
	6 mm below crest	1.05	0.38	0.28	2.25
	At apex	1.99	0.85	0.38	4.71

Left lateral incisor	2 mm below crest	1.05	0.47	0.12	2.87
	4 mm below crest	1.16	0.62	0.12	3.26
	6 mm below crest	0.98	0.58	0.12	3.86
	At apex	2.3	1.12	0.17	6.6

Left canine	2 mm below crest	1.06	0.45	0.04	2.86
	4 mm below crest	1.07	0.57	0.12	3.9
	6 mm below crest	0.94	0.55	0.16	3.25
	At apex	2.17	1.64	0	12.3

Abbreviation: SD, standard deviation.

**Table 6:** Descriptive statistics of palatal bone thickness by tooth type.

Tooth type	Distance	Mean	SD	Minimum	Maximum
Right canine	2 mm below crest	1.48	0.61	0.3	3.4
	4 mm below crest	2.14	1.04	0.43	5.7
	6 mm below crest	3.2	1.89	0.43	8.83
	At apex	8.32	2.99	0.6	17.46

Right lateral incisor	2 mm below crest	1.17	0.64	0.36	4.4
	4 mm below crest	1.85	1.29	0.54	8.22
	6 mm below crest	2.44	1.49	0.31	8.9
	At apex	5.68	2.38	1.19	12.7

Right central incisor	2 mm below crest	1.41	0.47	0.34	2.66
	4 mm below crest	2.15	0.83	0.72	4.55
	6 mm below crest	2.92	1.37	0.86	10.44
	At apex	7.38	2.14	1.7	12.6

Left central incisor	2 mm below crest	1.29	0.45	0.43	3.24
	4 mm below crest	2.07	0.81	0.7	4.23
	6 mm below crest	2.88	1.36	0.81	6.8
	At apex	7.8	2.32	3.43	12.85

Left lateral incisor	2 mm below crest	1.19	0.51	0	3.18
	4 mm below crest	1.75	0.77	0.5	4.7
	6 mm below crest	2.37	1.14	0.52	5.87
	At apex	5.66	1.83	1.44	10.5

Left canine	2 mm below crest	1.47	0.57	0.44	3
	4 mm below crest	2.14	1.02	0.33	5.89
	6 mm below crest	3.06	1.53	0.65	7.24
	At apex	8.56	3.1	2.84	16.76

Abbreviation: SD, standard deviation.

**Table 7:** Association between gender and sagittal root position (SRP) classification with tooth type.

Side	Tooth type	SRP class	Gender	*χ* ^2^ value	*p* value
			Female	Male	
			*n* (%)	*n* (%)	
Right	Canine	I	39 (50)	39 (50)	3.738
		II	3 (75)	1 (25)	
		III	0 (0)	2 (100)	
		IV	11 (61.11)	7 (38.89)	
	Lateral incisor	I	19 (38.78)	30 (61.22)	9.711
		II	21 (72.41)	8 (27.59)	
		III	2 (33.33)	4 (66.67)	
		IV	11 (61.11)	7 (38.89)	
	Central incisor	I	33 (47.14)	37 (52.86)	2.107
		II	9 (64.29)	5 (35.71)	
		III	0 (0)	0 (0)	
		IV	11 (61.11)	7 (38.89)	

Left	Central incisor	I	28 (42.42)	38 (57.58)	8.951
		II	12 (75)	4 (25)	
		III	0 (0)	1 (100)	
		IV	13 (68.42)	6 (31.58)	
	Lateral incisor	I	21 (46.67)	24 (53.33)	2.749
		II	16 (66.67)	8 (33.33)	
		III	2 (50)	2 (50)	
		IV	14 (48.28)	15 (51.72)	
	Canine	I	38 (50.67)	37 (49.33)	2.861
		II	3 (100)	0 (0)	
		III	1 (50)	1 (50)	
		IV	11 (50)	11 (50)	

**Table 8:** Distribution of sagittal root position (SRP) classification of teeth according to gender.

SRP class	Female	Male	Overall
	*n* (%)	*n* (%)	*n* (%)
I	178 (46.5)	205 (53.5)	383 (100)
II	64 (71.1)	26 (28.9)	90 (100)
III	5 (33.3)	10 (66.7)	15 (100)
IV	71 (57.3)	53 (42.7)	124 (100)
Overall	318 (52)	294 (48)	612 (100)

**Table 9:** Canine's labial and palatal alveolar bone thickness means by gender comparison.

Canine type	Distance	Gender				*p* value
		Female (106)		Male (98)		
		Mean	SD	Mean	SD	
Labial	2 mm from crest	0.98	0.39	1.19	0.59	0.004^*∗*^
	4 mm from crest	1.01	0.48	1.18	0.63	0.029^*∗*^
	6 mm from crest	0.87	0.47	1.07	0.57	0.006^*∗*^
	At the apex	2.11	1.59	2.22	1.15	0.590

Palatal	2 mm from crest	1.28	0.45	1.68	0.64	0.001^*∗*^
	4 mm from crest	1.84	0.94	2.46	1.03	0.001^*∗*^
	6 mm from crest	2.74	1.51	3.55	1.83	0.001^*∗*^
	At the apex	7.61	2.82	9.34	3.03	0.001^*∗*^

Abbreviation: SD, standard deviation.

*⁣* ^*∗*^Means statistically significant difference at 5% level of significance (*p*  < 0.05).

**Table 10:** Lateral incisors thickness means by gender comparison.

Lateral type	Distance	Gender				*p* value
		Female (106)		Male (98)		
		Mean	SD	Mean	SD	
Labial	2 mm from crest	0.96	0.42	1.2	0.5	0.001^*∗*^
	4 mm from crest	1.03	0.53	1.34	0.69	0.001^*∗*^
	6 mm from crest	0.92	0.49	1.12	0.65	0.015^*∗*^
	At the apex	2.29	1.18	2.39	1.1	0.518

Palatal	2 mm from crest	1.1	0.51	1.27	0.63	0.028^*∗*^
	4 mm from crest	1.63	0.95	1.98	1.15	0.018^*∗*^
	6 mm from crest	2.16	1.2	2.67	1.41	0.006^*∗*^
	At the apex	5.01	1.69	6.39	2.3	0.001^*∗*^

Abbreviation: SD, standard deviation.

*⁣* ^*∗*^Means statistically significant difference at 5% level of significance (*p*  < 0.05).

**Table 11:** Central incisors thickness means by gender comparison.

Central type	Distance	Gender				*p* value
		Female (106)		Male (98)		
		Mean	SD	Mean	SD	
Labial	2 mm from crest	1.02	0.29	1.1	0.35	0.08
	4 mm from crest	1.05	0.34	1.1	0.4	0.328
	6 mm from crest	0.96	0.35	1.03	0.41	0.209
	At the apex	1.82	0.85	2.08	0.83	0.029^*∗*^

Palatal	2 mm from crest	1.28	0.44	1.42	0.47	0.028^*∗*^
	4 mm from crest	1.9	0.69	2.34	0.89	0.001^*∗*^
	6 mm from crest	2.69	1.34	3.13	1.35	0.019^*∗*^
	At the apex	7.08	2.06	8.14	2.29	0.001^*∗*^

Abbreviation: SD, standard deviation.

*⁣* ^*∗*^Means statistically significant difference at 5% level of significance (*p*  < 0.05).

## Data Availability

The data that support the findings of this study are available from the corresponding author upon reasonable request.
